# Relationship between the Severity of Anxiety Symptoms, Aggression and Alcohol Consumption during the COVID-19 Pandemic Period

**DOI:** 10.3390/medicina57090959

**Published:** 2021-09-11

**Authors:** Szymon Florek, Paweł Dębski, Magdalena Piegza, Piotr Gorczyca, Robert Pudlo

**Affiliations:** Department of Psychiatry in Tarnowskie Gory, Faculty of Medical Sciences in Zabrze, Medical University of Silesia, 42-612 Tarnowskie Góry, Poland; pdebski@sum.edu.pl (P.D.); mpiegza@sum.edu.pl (M.P.); pgorczyca@sum.edu.pl (P.G.); rpudlo@sum.edu.pl (R.P.)

**Keywords:** COVID-19, pandemic, mental health, anxiety, aggression, alcohol drinking

## Abstract

*Background and objectives*: There have been many reports of mental health in the pandemic period. The research conducted so far has indicated an increase in the severity of anxiety and aggression and an escalation of alcohol consumption during the COVID-19 pandemic. The aim of this study was to determine the relationship between the severity of anxiety, the amount of alcohol consumed and the severity of aggression. *Materials and Methods*: A total of 538 Polish residents—413 women (76.77%) and 125 men (23.23%)—participated in the study via an online survey. It included socio-demographic parameters and psychological scales: AUDIT—to determine the model of alcohol consumption, GAD-7—to measure the severity of anxiety and the Buss–Perry Aggression Questionnaire. *Results*: There was a correlation between the severity of anxiety and generalized aggression, and its two components—anger and hostility—in the entire study group. Moreover, relations were found between the intensity of alcohol consumption and generalized aggression and its components—anger and verbal and physical aggression. Those relationships turned out to be gender specific. The increased anxiety intensity affected the pattern of alcohol consumption and the severity of aggression. *Conclusions*: Psychotherapeutic and psychoeducational interactions and some elements of psychiatric treatment should aim at reducing the severity of anxiety in society, and thus minimizing the health and social consequences—aggressive behaviour and excessive alcohol consumption.

## 1. Introduction

Over the years, many studies have been dedicated to the impact of various epidemics and pandemics on the mental health of large populations [1,2,3,4]. Their consequences were emphasized in the context of increasing the level of anxiety, the appearance of persistent insomnia or excessive alcohol consumption, as well as lowering the quality of life dynamics [5]. Currently, humanity is struggling with the pandemic caused by the SARS-CoV-2 virus. The media is constantly presenting the statistics and information about the next victims of the virus. Some researchers have postulated the identification of a new disease entity—pandemic acute stress disorder [6]—but the existing classifications allow for the identification and naming of numerous mental disorders, the severity of which may be exacerbated by the prevailing COVID-19 pandemic. According to the current scientific reports, in the time of the COVID-19 pandemic, an increase in mental disorders with the dominance of anxiety symptoms in various societies has been observed [7,8,9]. In the first of the mentioned studies, Hang Choi et al. indicated a significant increase in the percentage of people struggling with anxiety during the COVD-19 pandemic in Hong Kong [7]. A systematic review of the literature combined with a meta-analysis also identified this problem on a global scale, and one of the potential causes of increased anxiety given was the significant exposure of people to news about morbidity and mortality due to COVID-19 infection [8]. In addition, there have been studies that indicate the increased alcohol consumption [10,11,12,13,14] and increased tendency for aggression [15,16,17]. A study conducted during the COVID-19 pandemic among Australians showed that 1/4 of those surveyed increased their alcohol consumption [10]. In addition, a report by the Australian Foundation for Alcohol Research and Education suggested that 70% of Australians have consumed more alcohol than usual since the outbreak of the pandemic, and 28% of them drank to relieve anxiety and stress [11]. A study conducted in Poland showed that during the COVID-19 pandemic, almost 30% of respondents consumed risky amounts of alcohol, and over 14% increased their alcohol consumption in relation to the amounts consumed outside this period [12]. Also, Belgian and German studies have revealed that during the COVID-19 pandemic, there has been a phenomenon of increased alcohol consumption in these societies [13,14]. The limitations associated with the COVID-19 pandemic may both increase aggressive behaviour secondary to increased frustration [15] and predispose victims to persecution by perpetrators [16]. The studies conducted so far have also emphasized the fact that the deterioration of mental health is mainly caused by the exacerbations related to the pandemic or the death of a loved one, and not by the COVID-19 infection itself [18]. Similar conclusions can be observed in an American study conducted among young people, where 49% of respondents reported a high degree of loneliness, almost 80% declared significant symptoms of depression and 44% of participants reported binge drinking at least once a month [19]. However, these pieces of research are in the minority, and there have been no studies that could detect relationships between symptoms like anxiety, aggression and alcohol consumption.

Even Sigmund Freud and Melanie Klein—despite different approaches—emphasized the importance of fear as one of the primary phenomena [20]. Maze also introduced in his studies that there are expressions of primal urges that are responsible for the formation of fear [21]. However, it is worth taking a closer look at newer studies, such as that of Panskepp, who postulated that the fear system belongs to one of the seven main emotional systems of mammals [22]. These studies allow for the conclusion that anxiety is the basic phenomenon in relation to alcohol abuse and aggression. This has been confirmed by research conducted outside the pandemic period. One of those has shown that anxiety disorders may predispose people to increased alcohol consumption [23]. Another study that focused on social anxiety showed that this type of anxiety also contributed primarily to increasing aggression levels and less to increasing alcohol consumption [24]. It is also worth emphasizing that a high level of alcohol consumption often causes aggressive behaviour. This is probably due to the pharmacological effect of alcohol on the frontal areas of the brain and impaired processing of emotional stimuli, which may result in aggressive behaviour. These mechanisms, however, are complicated and require further explanation in future scientific research [25,26]. The authors of this study decided to check how the relations between these variables have been shaped in Polish society during the COVID-19 pandemic. Establishing this fact can help prevent, reduce and respond to the negative mental health effects of the pandemic.

The aim of this study was to determine the relationships between the severity of anxiety symptoms, the amount of alcohol consumed and the level of aggression. This research was conducted during the SARS-CoV-2 virus pandemic, in the period from 24 April 2020 to 8 May 2020 in a Polish population. At the beginning of the study—24 April 2020—381 new infections with the SARS-CoV-2 virus were recorded in Poland. A total of 40 people died on that day. At the end point of our study—on 8 May 2020—the Polish Ministry of Health issued the message informing the public of 319 new confirmed infections with the SARS-CoV-2 virus, while 21 people died on that day in Poland. Throughout the entire study, 4855 people in Poland were diagnosed with a new infection and 322 died. Those pieces of information were taken from the daily announcements of the Polish Ministry of Health. In our study, the authors examined whether, and how, the pandemic period influenced the relationship between the level of alcohol consumption, aggression and anxiety in society. The results were compared with studies conducted outside the pandemic period.

## 2. Material and Methods

This study was conducted via an online survey due to the constraints of the COVID-19 pandemic. Each respondent was informed about the purpose of the intended research and the manner of conducting it, and that the respondent may at any time refuse consent to participate in the study or withdraw it—even during the study. It was also emphasized that the processing of personal data would take place only to the extent necessary to conduct research, while maintaining all the rules of confidentiality and anonymity. Each person who entered the study was obliged to read and accept the instructions. In order to limit the duplication of answers and for follow up, the respondents were asked to provide e-mail addresses, which were removed from the database after the follow-up questionnaire. A total of 538 Polish residents participated in the study—413 women (76.77%) and 125 men (23.23%). Most of the respondents, as much as 68.03% (366), were 18–29 years old (y.o.), while 4.65% (25) of people were over 50 y.o. Overall, 19.52% (105) of the respondents lived in villages, while the rest lived in larger or smaller towns in Poland. It is also worth noting that 50.37% (271) of the respondents were people with higher education. The sociodemographic structure of the studied group did not differ from the previously published work [27]. This study was part of a larger project. The first part of the project was devoted to the pro-health role of ego-resiliency in relation to the severity of symptoms of anxiety and aggression and the role of resiliency in alcohol consumption [27].

In order to determine the model of alcohol consumption, the intensity of anxiety and aggression symptoms, an online study was created. It included respected psychological scales—AUDIT (Alcohol Use Disorders Identification Test), GAD-7 (Generalized Anxiety Disorder-7) and the Buss–Perry Aggression Questionnaire. An anonymous questionnaire was also constructed in order to determine the sociodemographic characteristics of the study population. There were also some questions relating to exclusion criteria. In our study, the exclusion criteria were any events that made major changes to the lives of respondents in the 12 months prior to the survey and psychiatry therapy within 6 months before research and age up to 18. The inclusion criteria involved all adult people who did not meet the exclusion criteria. The questionnaire was shared via online means of communication, including social media such as Facebook. It was dictated by the pandemic situation in Poland at that time and the nationwide lockdown introduced, which made it impossible to conduct the study in any other way. The study took place after obtaining the approval of The Bioethical Committee operating at the Medical University of Silesia. The participants of the study were informed about the purpose of conducting this study, as well as about the possibility of withdrawing from participation in the research project at any stage. They were also familiarized with the principles of anonymity during the analysis of the collected results.

### 2.1. AUDIT Test

The Polish version of the international AUDIT test [28] was used to measure the level of alcohol dependence. The questionnaire consists of 10 questions. The test assumes the possibility of answering on a five-point scale, and each question can be obtained from 0 to 4 points. The total test score is obtained by adding up the points for each answer. Cronbach’s alpha in our study was 0.79.

### 2.2. GAD-7 Scale

In order to determine the intensity of the anxiety level, the scale developed by R.L. Spitzer, J.B.W. Williams, K. Kroenke and colleagues was used [29]. In the Polish language version, it contains 7 questions, and for each of them the respondent may receive from 0 to 3 points. The scale result is the sum of the points obtained by the examined person. The respondents answer the questions included in the scale with based on their lives in the 2 weeks preceding the survey. The usefulness of the original tool was assessed by correlating the GAD-7 results with the diagnoses of mental health professionals. The original tool has satisfactory psychometric properties. Its sensitivity is 89% and its specificity is 82%. Cronbach’s alpha in our study equalled 0.87.

### 2.3. Buss–Perry Aggression Questionnaire

In order to determine the level of aggression, the scale, originally designed by A.H. Buss and M. Perry, was applied [30]. The respondents filled in the Polish version of this scale created by the Amity Institute [31]. It consists of 29 statements. Each of them is assessed on a five-point Likert scale, in which it is possible to obtain from 1 to 5 points. Scores on this scale are obtained by summing the individual points, taking into account two questions for which the score is counted inversely. In addition, the results of this scale involve various components of generalized aggression, which include physical aggression, verbal aggression, anger and hostility. The adaptive studies revealed the four-factor structure of the scale. Statistical analyses showed that the subscales of the tool are characterized by constancy between 0.72 and 0.80, and theoretical validity studies have indicated that the psychometric properties of the tool are satisfactory [31]. Cronbach’s alpha in our study for the main scale was 0.88.

### 2.4. Statistical Analysis

Statistical analyses were carried out using Excel 2016 and Statistica version 13.3. The Shapiro–Wilk test was used to assess the normality of distributions. Spearman’s rank correlation coefficient was used for the correlation matrix. The Mann–Whitney U test was used to analyse the intergroup differences. Linear regression models were also prepared to capture the role of anxiety in the severity of aggression and alcohol use. For this purpose, the regression procedure was used. The internal consistency of scales used was estimated using the Cronbach’s alpha coefficient. Harman’s single factor test was used to evaluate the common method bias. The obtained result indicates that the one-factor assumption explains 17.806% of the variance of all test items. It can, therefore, be concluded that the study does not have a serious CMB (common method bias) burden. A significance level of *p* < 0.05 was adopted in the statistical procedures.

## 3. Results

The performed statistical analysis for the entire study population showed a relationship between the level of anxiety and generalized aggression, as well as its two components—anger and hostility. The relationship between the severity of anxiety and anger and hostility turned out to be positive and moderate. Moreover, significant positive relationships were observed between the intensity of alcohol consumption and the level of generalized aggression and its components—anger, verbal and physical aggression. The described dependencies are presented in Table 1.

The prepared regression model involving the entire study population showed a significant, positive effect of anxiety intensity and physical aggression and anger as parts of generalized aggression on the level of alcohol consumption (Table 2).

A positive influence of anxiety on the development of generalized aggression was also observed among the examined respondents (Table 3).

Statistically significant results were also obtained by determining the relationship between the examined symptoms depending on gender. In the group of women, a positive correlation was found between the severity of anxiety and generalized aggression, anger and hostility—similarly to the analysis for all respondents. Significant positive relationships were also observed between the model of alcohol use in women and the level of generalized aggression and all four of its components. The described dependencies are presented in Table 4.

A positive correlation between the level of anxiety and the intensity of alcohol consumption was found in the group of men, which was not observed in the group of women. Moreover, anxiety in men turned out to be positively and moderately related to generalized aggression and its two components—anger and hostility. This relationship turned out to be slightly stronger than in the case of women. However, no significant associations were found between alcohol consumption by men and aggression (Table 5).

In the comparative analysis relevant to gender, a significantly higher level of anxiety and two components of aggression (anger and hostility) were observed in the group of examined women. Among the men, compared to women, the intensity of alcohol consumption and physical aggression turned out to be significantly higher. The differences of the compared variables in relation to gender are presented in Table 6.

## 4. Discussion

The studies published so far indicate that the pandemic has significantly increased the level of anxiety in the general population [5]. Women, in particular, are at risk of experiencing severe symptoms of anxiety related to the pandemic [32,33,34]. The results obtained by the authors of this study also confirm a higher level of anxiety in women than men, which may be related to many different factors, such as information about the pandemic from the media or cases of illness among friends or family. Moreover, among the surveyed women, significantly higher rates of anger and hostility, two of the four components of generalized aggression, were observed. This is consistent with the majority of global observations [35,36]. However, a study conducted during the COVID-19 pandemic in China showed the same severity of anxiety in both women and men [37], which may contribute to the consideration of intercultural differences or specific situations regarding pandemic and lockdowns. The study on the level of anxiety conducted in Ireland, in which the GAD-7 scale was also used, showed that in the early period of quarantine due to the COVID-19 pandemic, the highest scores on this scale were obtained by young people who were women and citizens struggling with loss of earnings due to restrictions. Other factors contributing to the increase in the anxiety score were: having a confirmed or suspected case of COVID-19, knowing a loved one with a confirmed or suspected case of COVID-19, and a moderate or high level of perceived risk of becoming infected with COVID-19 within the next month [38]. It is important to conduct other studies which can explain how men and women experience anxiety during a pandemic.

Similar statistical data were obtained in a study of a group of adolescents, where it was revealed that the level of perceived anxiety directly correlated with generalized aggression and its components, and a group of teenage girls were more susceptible to experiencing anxiety [36]. In published articles concerning aggression in the time of the COVID-19 pandemic, the topic of physical violence in the family has been discussed [39,40]. The results contained in the studies are consistent with those obtained in presented research—physical aggression was particularly intense in the group of male respondents. In conclusion, the increased sense of fear could cause an expression of aggression in the form of anger and hostility among women, while among men, it could mainly be expressed by physical aggression. It is worth emphasizing that there are studies pointing to the relationship between the COVID-19 pandemic and an increased amount of aggressive behaviour on the Internet [41]. Another study also found an increase in the level of aggression in society during the lockdown caused by the COVID-19 pandemic. It was particularly visible in the US in June and July, when it was decided to extend the existing restrictions [42].

In the conducted study, the level of alcohol consumption in the respondents was also checked. Other studies assessing the amount of alcohol consumed during the COVID-19 pandemic showed an increase in the consumption of this type of drink. The authors also point out the need to monitor the amount of alcohol sold in stores [43,44]. As Laska suggested, the problem might have negative effects, especially in people who require hospitalization in detoxification units, which could temporarily be transformed into COVID-19 units [45].

A particular value of our research is the identification of anxiety as a factor impacting on aggression and excessive alcohol consumption [46]. These results correspond to some concepts of addiction pathogenesis, which emphasize the high level of anxiety in some groups of addicted people [47,48]. It is also known that high levels of alcohol consumption may increase the experience of anxiety, as well as aggression [46,49]. Moreover, studies have reported that increased alcohol consumption (which increases the level of aggression and anxiety), in some cases, is associated with an existing mental disorder [50]. Research on soldiers has shown that both ASD (Acute Stress Disorder) and PTSD (Post-Traumatic Stress Disorder) predispose to increased alcohol consumption [51,52]. It is worth mentioning that some scientists believe that one type of mental disorder associated with the COVID-19 pandemic may be a specific form of ASD [6].

Our research is embedded in the realities of the pandemic and has been conducted with a large group of respondents. Considering the age and education structure of the respondents, it can be said with a high degree of certainty that the observed relationship has been proven for well-educated people between 18 and 49 years old. It is a significant group of people with maximum professional activity and a significant—perhaps dominant—influence on the functioning of society, so the high level of aggression and alcohol use in this group has far-reaching social and family consequences. Hence, there is an urgent need to develop interventions reducing the intensity of anxiety in this subpopulation, which should result in reducing the social effects of aggression and addiction. Our results suggest that anti-anxiety interventions may have an advantage over those aimed directly at preventing aggression and alcohol abuse.

The models of these interventions are beyond the scope of this study, however, it is worth remembering that—as shown by several reports—excessive exposure to information from various media sources increases the intensity of anxiety [53,54,55]. It has also been proven that focusing on the disease caused by the SARS-CoV-2 virus is the greatest risk of increasing the severity of anxiety [49]. Perhaps, it is necessary to rethink and modify information policies. It is natural that the media seek sensation and to astonish with alarming information, but today it seems necessary to constantly and regularly organize these reports by calmly, factually and credibly describing the reality in all its complexity. Perhaps this should be the role of publicly financed media in times of the pandemic.

It is worth mentioning the limitations of this study. First of all, there were more young than old people among the respondents. Perhaps, it is consequence of the form of this research, because it was provided only as an online survey. The second limitation is according to the size of the city and the level of education. In our study, there were more people with higher education than without. Similarly, there were more people from big cities than small cities and the countryside. We think that these differences come from Internet access in towns and villages. However, this dependence could be result of the degree of understanding of the need to complete the survey. Conducting scientific research only via Internet may also involve some distorting of reality, and thus false answers of the respondents. This form of research was dictated by the conditions connected with lockdown in Poland. It is also worth paying attention to the fact that, in this study, the differences of the examined factors were analysed only in terms of gender. This may cause some confusion due to the heterogeneity of the male and female groups. Such an approach from the authors resulted from the desire to present the data in a simple and legible manner. An in-depth analysis of the individual parameters within each group could complicate this considerably, and the length of this article would be beyond the scope of the journal.

Due to the above limitations of the study, it seems justified to conduct similar studies in different age groups. It may also be valuable to conduct it through direct contact with respondents. Our study is part of a larger project that involves collecting follow-up data and analysing it compared to the data presented here.

## 5. Conclusions

Increased intensity of anxiety correlates with higher alcohol consumption and the occurrence of aggressive behaviour. The period of the pandemic has not disturbed the relationship between them.

Anxiety-reducing reactions might help to reduce alcohol consumption and the risk of aggression, thereby minimizing the global social and health impact of a pandemic.

There is a need for more research comparing the levels of anxiety, aggression and alcohol consumption in society during and outside the pandemic.

It is justified to carry out further research indicating the most beneficial forms of reducing anxiety, both across the population and in individual people. The results of previously conducted studies may be out of date due to significant limitations in interpersonal communication in the time of the COVID-19 pandemic.

## Figures and Tables

**Table 1:** Relationships between the severity of anxiety, the level of alcohol consumption and aggression.

*n* = 538	Anxiety	Alcohol	Generalized Aggression	Physical Aggression	Verbal Aggression	Anger	Hostility
Anxiety	1.000	0.060	0.275 *	0.014	0.071	0.341 *	0.312 *
Alcohol		1.000	0.166 *	0.138 *	0.137 *	0.150 *	0.073
Generalized aggression			1.000	0.675 *	0.633 *	0.840 *	0.772 *
Physical aggression				1.000	0.375 *	0.438 *	0.297 *
Verbal aggression					1.000	0.433 *	0.308 *
Anger						1.000	0.538 *
Hostility							1.000

* Statistically significant result at *p* < 0.05; SE—standard error.

**Table 2:** Model of regression of the level of alcohol consumption in relation to the intensity of symptoms of anxiety and parts of aggression.

Predictor	b	b SE	±95% CI for b	Βeta	Beta SE	T	±95% CI for Beta	*p*
Constant	0.660	0.804	−0.920–2.240	---	---	0.821	---	0.412
Anxiety	0.084	0.035	0.016–0.153	0.111	0.046	2.435	0.021–0.201	0.015 *
Physical aggression	0.131	0.036	0.059–0.202	0.175	0.049	3.583	0.079–0.270	<0.001 *
Verbal aggression	0.019	0.052	−0.083–0.122	0.018	0.048	0.372	−0.076–0.112	0.710
Anger	0.080	0.038	0.005–0.155	0.119	0.056	2.109	0.008–0.230	0.035 *
Hosility	−0.044	0.031	−0.105–0.018	−0.071	0.050	−1.405	−0.169–0.028	0.161

* Statistically significant result at *p* < 0.05; SE—standard error; CI—confidence interval. Corr. R-squared = 0.745; F(5.532) = 8.566; *p* < 0.001; error of estimation = 3.870.

**Table 3:** Regression model of generalized aggression towards severity of anxiety.

Predictor	b	b SE	±95% CI for b	Βeta	Beta SE	±95% CI for Beta	t	*p*
Constant	63.896	1.142	61.653–66.138	---	---		55.974	<0.001 *
Anxiety	0.848	0.127	0.599–1.097	0.278	0.041	0.196–0.359	6.691	<0.001 *

* Statistically significant result at *p* < 0.05; SE—standard error; CI—confidence interval. Corr. R-squared = 0.075; F(1.536) = 44.771; *p* < 0.001; error of estimation = 15.442.

**Table 4:** Relationships between the severity of anxiety, the level of alcohol dependence and aggression in women.

Women *n* = 413	Anxiety	Alcohol	Generalized Aggression	Physical Aggression	Verbal Aggression	Anger	Hostility
Anxiety	1.000	0.053	0.258 *	0.024	0.094	0.308 *	0.302 *
Alcohol		1.000	0.176 *	0.113 *	0.126 *	0.202 *	0.101 *
Generalized aggression			1.000	0.693 *	0.673 *	0.852 *	0.780 *
Physical aggression				1.000	0.372 *	0.479 *	0.350 *
Verbal aggression					1.000	0.525 *	0.349 *
Anger						1.000	0.534 *
Hostility							1.000

* Statistically significant result at *p* < 0.05.

**Table 5:** Relationships between the severity of anxiety symptoms, the level of alcohol dependence and aggression in men.

Men *n* = 125	Anxiety	Alcohol	Generalized Aggression	Physical Aggression	Verbal Aggression	Anger	Hostility
Anxiety	1.000	0.224 *	0.323 *	0.155	0.019	0.335 *	0.325 *
Alcohol		1.000	0.143	0.111	0.134	0.104	0.058
Generalized aggression			1.000	0.689 *	0.500 *	0.813 *	0.752 *
Physical aggression				1.000	0.379 *	0.465 *	0.236 *
Verbal aggression					1.000	0.174	0.208 *
Anger						1.000	0.512 *
Hostility							1.000

* Statistically significant result at *p* < 0.05.

**Table 6:** Sex differences in terms of the studied variables.

Variable	Women *n* = 413			Men *n* = 125			Cohen’s d	Z	*p*
	Average	SD	Median	Average	SD	Median			
Anxiety	7.855	5.187	7.000	5.552	5.131	4.000	0.4	4.779	<0.001 *
Alcohol	3.777	3.493	3.00	5.640	5.119	5.000	0.5	−4.417	<0.001 *
Generalized aggression	70.395	16.229	68.000	69.128	15.506	67.000	0.1	0.724	0.469
Physical aggression	15.729	5.189	15.000	17.776	5.594	17.000	0.4	−3.910	0.000 *
Verbal aggression	14.521	3.711	14.000	14.832	3.587	15.000	0.0	−0.945	0.344
Anger	18.719	5.888	18.000	16.488	5.844	16.000	0.4	3.474	0.001 *
Hostility	21.426	6.449	22.000	20.032	6.244	20.000	0.2	2.309	0.021 *

* Statistically significant result at *p* < 0.05, Z—statistical Mann–Whitney U value.

## Data Availability

The data presented in this study are available on request from the corresponding author. The data are not publicly available due to private survey research.
